# Serial Analysis of Gene Mutations and Gene Expression during First-Line Chemotherapy against Metastatic Colorectal Cancer: Identification of Potentially Actionable Targets within the Multicenter Prospective Biomarker Study REVEAL

**DOI:** 10.3390/cancers14153631

**Published:** 2022-07-26

**Authors:** Jörg Kumbrink, Lisa Bohlmann, Soulafa Mamlouk, Torben Redmer, Daniela Peilstöcker, Pan Li, Sylvie Lorenzen, Hana Algül, Stefan Kasper, Dirk Hempel, Florian Kaiser, Marlies Michl, Harald Bartsch, Jens Neumann, Frederick Klauschen, Michael von Bergwelt-Baildon, Dominik Paul Modest, Arndt Stahler, Sebastian Stintzing, Andreas Jung, Thomas Kirchner, Reinhold Schäfer, Volker Heinemann, Julian W. Holch

**Affiliations:** 1 Institute of Pathology, Faculty of Medicine, Ludwig-Maximilians-University of Munich (LMU), 80337 Munich, Germany; lisa.bohlmann@med.uni-muenchen.de (L.B.); peilstoecker.daniela@web.de (D.P.); lipan90med@gmail.com (P.L.); harald@bartsch.bayern (H.B.); jens.neumann@med.uni-muenchen.de (J.N.); frederick.klauschen@med.uni-muenchen.de (F.K.); andreas.jung@med.uni-muenchen.de (A.J.); thomas.kirchner@med.uni-muenchen.de (T.K.); 2 Partner Site Munich, German Cancer Consortium (DKTK), 80336 Munich, Germany; michael.bergwelt@med.uni-muenchen.de (M.v.B.-B.); volker.heinemann@med.uni-muenchen.de (V.H.); julian.holch@med.uni-muenchen.de (J.W.H.); 3 Partner Site Berlin, German Cancer Consortium (DKTK), 10117 Berlin, Germany; soulafa.mamlouk@charite.de (S.M.); dominik.modest@charite.de (D.P.M.); arndt.stahler@charite.de (A.S.); sebastian.stintzing@charite.de (S.S.); reinhold.schaefer@charite.de (R.S.); 4 German Cancer Research Center (DKFZ), 69120 Heidelberg, Germany; 5 Institute of Pathology, Charité—Universitätsmedizin Berlin, 10117 Berlin, Germany; 6 Institute of Medical Biochemistry, University of Veterinary Medicine Vienna, 1210 Vienna, Austria; torben.redmer@vetmeduni.ac.at; 7 Klinik und Poliklinik für Innere Medizin III, Klinikum Rechts der Isar, Technical University of Munich, 81675 Munich, Germany; sylvie.lorenzen@mri.tum.de; 8 School of Medicine, Technical University of Munich, 81675 Munich, Germany; hana.alguel@mri.tum.de; 9 Comprehensive Cancer Center Munich, Klinikum Rechts der Isar, Technical University of Munich, 81675 Munich, Germany; 10 Department of Medical Oncology, West German Cancer Center, University Hospital Essen, 45147 Essen, Germany; stefan.kasper-virchow@uk-essen.de; 11 Steinbeishochschule Berlin, 12489 Berlin, Germany; dirk.hempel@onko-medeor.de; 12 Steinbeis Transfer Institute Clinical Hematology-Oncology, 86609 Donauwörth, Germany; 13 VK&K Studien GbR, 84036 Landshut, Germany; info@vehling-kaiser.de; 14 Department of Medicine III, University Hospital, LMU Munich, 81377 Munich, Germany; marlies.michl@med.uni-muenchen.de; 15 Comprehensive Cancer Center, University Hospital, LMU Munich, 81377 Munich, Germany; 16 Department of Hematology, Oncology and Cancer Immunology (CCM), Charité—Universitätsmedizin Berlin, 10117 Berlin, Germany; 17 Charité Comprehensive Cancer Center, Charité—Universitätsmedizin Berlin, 10117 Berlin, Germany

**Keywords:** metastatic colorectal cancer, next generation sequencing, gene expression signature, biomarker, liquid biopsy, secondary resistance, therapeutic target

## Abstract

**Simple Summary:**

The emergence of resistant cells remains a major obstacle for chemotherapy treatment of metastatic colorectal cancers. Improvement of the therapeutic response requires a thorough understanding of the mechanisms of resistance as well as informative biomarkers. In the REVEAL study, we have systematically compared the mutational patterns and expression profiles of primary tumor specimens before and after first-line chemotherapy treatment in the metastatic situation. In addition, we analyzed liquid biopsies pre, during, and after treatment. Alterations in gene expression appeared as the major drivers of chemotherapy resistance. We identified a gene expression signature differentiating primary tumors and metastases and validated this signature in two independent patient cohorts. Moreover, we evaluated the expression of two signature genes, *SFRP2* and *SPP1*, as prognostic and potentially druggable biomarkers.

**Abstract:**

Most metastatic colorectal cancer (mCRC) patients succumb to refractory disease due to secondary chemotherapy resistance. To elucidate the molecular changes associated with secondary resistance, we recruited 64 patients with mCRC and hepatic metastases before standard first-line chemotherapy between 2014 and 2018. We subjected DNA from primary tumor specimens (P), hepatic metastasis specimens after treatment (M), and liquid biopsies (L) taken prior to (pre), during (intra), and after (post) treatment to next generation sequencing. We performed Nanostring expression analysis in P and M specimens. Comparative bioinformatics and statistical analysis revealed typical mutational patterns with frequent alterations in *TP53*, *APC*, and *KRAS* in P specimens (*n* = 48). P and pre-L (*n* = 42), as well as matched P and M (n = 30), displayed a similar mutation spectrum. In contrast, gene expression profiles classified P (*n* = 31) and M (*n* = 23), distinguishable by up-regulation of immune/cytokine receptor and autophagy programs. Switching of consensus molecular subtypes from P to M occurred in 58.3% of cases. M signature genes *SFRP2* and *SPP1* associated with inferior survival, as validated in an independent cohort. Molecular changes during first-line treatment were detectable by expression profiling rather than by mutational tumor and liquid biopsy analyses. *SFRP2* and *SPP1* may serve as biomarkers and/or actionable targets.

## 1. Introduction

Colorectal cancer (CRC) is the third leading cause of cancer worldwide with 1.93 million people affected globally, accounting for 10% of all cancer deaths [1]. The high mortality is explained in part by the fact that nearly 20% of patients present with de novo metastatic disease, and 25–30% of patients with stage II/III disease have a recurrence within five years of a curative intended surgery [2]. A major obstacle in the treatment of metastatic CRC (mCRC) is the development of drug resistance during systemic treatment [3]. Median overall survival (OS) exceeding 30 months has been reached in selected patients [4,5,6] following the introduction of modern chemotherapy in combination with monoclonal antibodies such as bevacizumab, cetuximab and panitumumab targeting vascular endothelial growth factor (VEGF) and epithelial growth factor receptor (EGFR), respectively. However, a high mortality rate with a 5-year survival of only 12% indicates the need for further understanding therapy resistance and metastatic mechanisms, as well as for identifying novel prognostic biomarkers and potential therapeutic targets [7].

Previous studies have addressed the mutational landscape in primary and metastatic CRCs and found that genomes of metastases are essentially not different from those of primary tumors. [8,9]. Only a few studies investigated gene expression profiles in primary tumors and metastases utilizing distinct comparative models to identify prognostic metastasis signatures and biomarkers [8,9,10,11]. In these analyses, transcriptomic differences in cellular programs, such as downmodulation of epithelial–mesenchymal transition (EMT) and differential expression of a few single genes, were described [9,10,11]. However, most of these investigations were performed on samples of primary tumors and metastases; as both were obtained prior to any chemotherapeutic therapy, they thus do not reflect the massive influence of cytotoxic agents on the expression levels of numerous genes in metastases.

Recently, circulating tumor DNA (ctDNA) has emerged not only as a promising noninvasive biomarker but also as a clinical tool for therapeutic and relapse monitoring. Different approaches of ctDNA analysis in pre- and post-operative specimens were investigated in solid tumors including CRCs [12,13,14,15,16,17], and ctDNA analysis also detected minimal residual disease and predicted recurrence in patients with stage I-III colon cancer [17,18]. Personalized ctDNA deep sequencing of stage I to III CRCs showed the potential of ctDNA analysis to change post-operative management and early relapse detection [15]. The value of ctDNA analysis in mCRCs, e.g., for RAS and BRAF typing, was investigated in only a few studies so far [16,19].

Here, we report results from the prospective observational biomarker study REVEAL (ReEVAluation of Liver metastasis) in patients with previously untreated mCRC. We investigated tumor and liquid biopsies before, during and after standard fist-line chemotherapy. By next-generation sequencing (NGS) of 100 CRC-specific genes using a customized gene panel [20], we explored the potential of ctDNA detection for tracking tumor mutations under first-line treatment as well as compared mutational patterns in primary tumor before and liver metastasis after first-line treatment. Furthermore, we explored the gene expression of 770 cancer-associated genes in untreated primary tumors and metastatic tissue after first-line chemotherapy utilizing the Nanostring system.

## 2. Materials and Methods

### 2.1. Study Design, Patients and Samples

The REVEAL study (ReEVAluation of Liver metastases) is a prospective, multicenter, observational biomarker study for the indication of mCRC. Patients were recruited from hospitals and private practices in Germany (Table S1). Inclusion criteria: Age ≥ 18 y; ECOG (Eastern Cooperative Oncology Group) performance status 0–1; stage IV, histologically confirmed adenocarcinoma of the colon or rectum; presence of measurable liver metastases according to RECIST (Response Evaluation Criteria in Solid Tumors) version 1.1; intention to initiate standard mCRC chemotherapy according to physician’s choice; recruitment irrespective of *RAS* or *BRAF* status; white blood cell count ≥ 3.0 × 10^9^ cells/L; neutrophils ≥ 1.5 × 10^9^ cells/L; platelets ≥ 100 × 10^9^/L; hemoglobin ≥5.6 mmol/L (corresponding to 9.0 g/dL); serum bilirubin ≤ 1.5 × upper limit of normal (ULN); alanine aminotransferase and aspartate aminotransferase ≤ 2.5 × ULN or ≤5 × ULN in the presence of liver metastases; serum albumin ≥ 2.5 g/dL. Exclusion criteria: previous CRC chemotherapy, excluding adjuvant therapy completed at least 6 mo before trial enrolment; severe bleeding within past 6 mo and any severe coagulopathy; myocardial infarction within past 6 mo, congestive heart failure (New York Heart Association classification > 2); serious non-healing wounds and a history of secondary malignancy within the past 5 y.

Tissue specimens from primary tumor biopsied were obtained before study entry. In case of progressive disease (PD) upon first-line chemotherapy, another biopsy of the colorectal liver metastasis was intended according to informed consent to study procedures before enrolment. Of course, refusal and study withdrawal by the patient was possible at any time point. In case of secondary hepatic resection, tumor specimens of the hepatic metastasis were retrieved. Before (pre-), during (intra-) and after (post-) first-line systemic treatment, serial blood samples to evaluate ctDNA were taken (liquid biopsies). During treatment, blood samples were taken every 4 weeks. Circulating free DNA (cfDNA) was extracted from blood serum (2–6 mL). Serum was prepared 1–7 h after blood draw and stored in polypropylene tubes at −20 °C until cfDNA extraction.

The trial was done in compliance with the Declaration of Helsinki. The protocol was approved by local ethics committees of all participating centers.

### 2.2. Histopathological Samples

Histopathological diagnosis and classification was reviewed for every available tumor specimen at the accredited Institute of Pathology of the University of Munich (Germany). In all cases, histopathological grade was confirmed by an experienced pathologist. Sections from formalin-fixed paraffin-embedded (FFPE) tissue samples were prepared followed by hematoxylin–eosin staining of one slide. Areas with a minimum percentage of 50% tumor cells were microdissected from subsequent unstained sections and used for DNA and RNA preparation. Normal tissue samples were taken at a minimum distance of 0.5 cm from the tumor site.

### 2.3. DNA Extraction and NGS Analyses

Genomic DNA (gDNA) was isolated from FFPE tissue sections using the Generead kit (Qiagen, Hilden, Germany), and cfDNA was extracted with the QIAmp Circulating Nucleic Acid Kit (Qiagen) following the vendor’s recommendations and as described previously [21]. The gDNA and cfDNA were used as template for targeted next-generation-sequencing (NGS) using the customized CRC sequencing panel, covering 100 frequently mutated genes with 784 amplicons (covering 21,000 COSMIC (Catalogue of Somatic Mutations In Cancer) mutations) [20]. Libraries were prepared using 20 ng DNA with the Ampliseq Library 2.0 kit and analyzed on the IonTorrent PGM (Personal Genome Machine; gDNA) or Ion S5 (Ion GeneStudio S5 prime; cfDNA) platforms (both Thermo Fisher Scientific, Darmstadt, Germany). The experimental procedures were conducted according to the manufacturer’s manual. Briefly, DNA concentration was measured using the Qubit3 Fluorimeter (Thermo Fisher Scientific). The concentration of amplifiable gDNA was quantified using a TaqMan RNaseP Detection Reagents Kit (Thermo Fisher Scientific). The cutoff for further processing was a minimum of 1 ng/µL RNaseP. Groups of two tumor samples or six health control samples (gDNA) or eight (cfDNA) libraries were transferred into the IonChef pipetting station for clonal amplification by emulsion PCR and Ion-316 Chip or Ion-550 Chip loading followed by sequencing on the PGM or S5, respectively. Sequencing data were aligned to the human reference genome hg19 using Torrent Suite™ (v5.8). Analysis of the NGS data was performed with SoFIA [22] (gDNA) or with Ion Reporter™ v5.10 (cfDNA) software, the Integrated Genomics Viewer (IGV, Broad Institute), and an in-house calling tool for the identification of tumor variants (SNV, single nucleotide variants; Insertions; Deletions) and the tumor genetic evaluation of the identified alterations. The following sequencing quality metrics were applied: 1) average base coverage depth gDNA ≥ 500 or cfDNA ≥ 5000; 2) percent reads on target ≥ 70%; or 3) uniformity of base coverage gDNA ≥ 85% or cfDNA ≥ 70%. Sequencing quality metrics for each sample are presented in Table S2. Patient-specific SNPs (single nucleotide polymorphisms) were utilized to confirm identity of the matched samples and for removal of healthy tissue ‘normal’ SNPs. The clinical relevance of the identified tumor variants was evaluated based on the ClinVar [23], COSMIC [24], and Varsome [25] databases/tools. Only likely pathogenic, pathogenic, and VUS (variant of unknown significance with a prediction trend of being likely pathogenic) variants with allele frequencies ≥ 5% (gDNA) or ≥0.5% (cfDNA) were reported.

### 2.4. RNA Extraction and Expression Analysis (NanoString^®^ nCounter Assay)

Total RNA was extracted from six to twelve sections of FFPE tissue sections using the RNeasy FFPE Kit (Qiagen, Hilden, Germany) according to the manufacturer’s instructions. RNA yield and purity were assessed using the NanoDrop ND-1000 spectrophotometer (NanoDrop Technologies, Rockland, ME, USA). A 260/280 optical density ratio within 1.7–2.3 and a minimal RNA concentration of 10 ng/µL were required for further processing. The mRNA expression was measured with the NanoString nCounter FLEX Analysis System (NanoString Technologies, Seattle, WA, USA) using 100 ng of total RNA and the pan cancer pathway panel (770 genes). The nCounter CodeSet was hybridized to total RNA for 18 h at 65 °C and nCounter Prep Station loading, and expression quantification with the nCounter Digital Analyzer was performed as recommended by the manufacturer.

### 2.5. RNA Expression Quality Control (QC) and Filtering of the Data

QC was performed with default nSolver v4.0 software settings and by analyzing the positive/negative controls, reference genes, total counts, and binding densities in each sample. Reference genes with an expression variation within all samples > 100% and expression values below the limit of detection (positive control E) were excluded. Reference genes for data normalization were *AMMERCR1L*, *C10orf76*, *CNOT4*, *COG7*, *DDX50*, *DHX50*, *DHX16*, *EDC3*, *EIF2B4*, *FCF1*, *FTSJ2*, *MRPS5*, *MTMR14*, *PIAS1*, *PIK3R4*, *SAP130*, *SF3A3*, *SWLC4A1AP*, *TLK2*, *TMUB2*, *TTC31*. nSolver as well as Box plot and similarity matrix analysis (MKmisc package [26]) in R (Figure S1) were used for QC after normalization. Genes with an average expression within all tumor samples < 30 and the lowest varying 10% of genes were excluded from further analyses. Log_2_ expression levels of leftover genes in all patient samples are presented in Table S3.

### 2.6. Statistical Analysis of the Filtered RNA Expression Data

The expression data were analyzed utilizing nSolver v4.0 (NanoString), R studio 3.5.3/4.0.2 [27], SPSS 22.0 (SPSS, Chicago, IL, USA), GraphPad PRISM 8 (GraphPad Software, Inc., San Diego, CA, USA), Perseus [28], MR4Cancer [29], GSEA [30] (gene set enrichment analysis), and STRING [31] (Search Tool for the Retrieval of Interacting Genes/Proteins) software. Validation datasets were downloaded from GEO [32] (Gene Expression Omnibus). Normal distribution of the datasets was confirmed by quantile–quantile (q-q) plot in R (data not shown). Expression changes and differentially expressed genes (DEGs) were identified by moderated *t*-test with limma (linear models for microarray) in R [33]. Adjusted P values/false discovery rates (FDR) were calculated using Benjamini and Hochberg correction [34]. Unsupervised hierarchical clustering/heat maps were performed with the ComplexHeatmap [35] and volcano plots with EnhancedVolcano packages in R. PCA (Principal Component Analysis) were generated in Perseus. GO (gene ontology) analyses were performed with MR4Cancer (Colon adenocarcinoma), GSEA (Hallmark and C1-C7 datasets) and STRING (FDR = stringency 5%; minimum required interaction score: 0.4). CMS (consensus molecular subtype) classification/prediction (nearest template prediction) was conducted with CMScaller R package [36].

Overall survival (OS) was defined as the time from start of chemotherapy to death due to any cause. Distributions of this time-to-event variable was estimated with the Kaplan–Meier method and compared with logrank test. The effect of molecular markers was estimated with the Cox proportional hazards model. To identify an optimized threshold value to discriminate high from low expression, the maximum of sensitivity and specificity of logarithmic expression data was calculated using a receiver operator characteristic (ROC) model (R package ggplot2, R version 4.0.2). Metastasis prediction was computed by using multivariate logistic regression to obtain coefficients for each gene. Coefficients were multiplied with the continuous expression values for the corresponding gene and subsequently added. To determine how well the metastasis prediction model discriminates primary tumor and metastasis, ROC analysis was performed. All *p*-values < 0.05 (two-sided) were regarded significant.

## 3. Results

### 3.1. Study Design and Population Demographics

**Figure 1:**
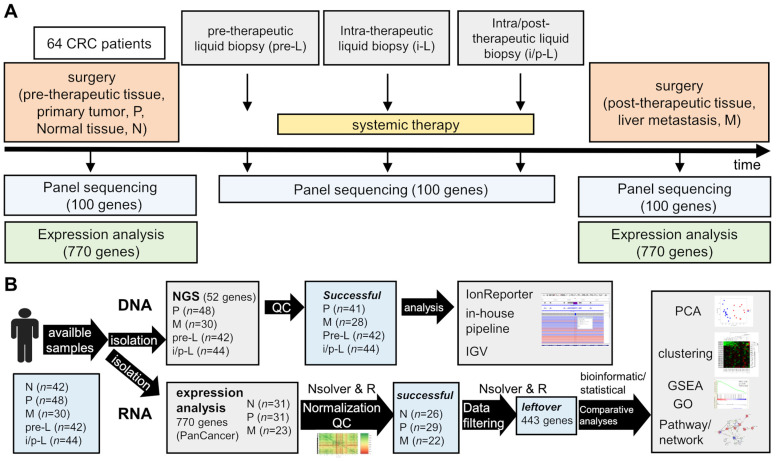
Design (**A**) and workflow of the data analyses (**B**) of the REVEAL study (M, post-therapeutic tissue, liver metastasis; N, normal tissue in the vicinity of the primary tumor; P, pre-therapeutic primary tumor; i-L, intra-therapeutic liquid biopsy; i/p-L, intra/post-therapeutic liquid biopsy); GO, gene ontology analysis; GSEA, gene set enrichment analysis; IGV, integrative genomics viewer; PCA, principal component analysis; QC, quality control.

Between 2014 and 2018, altogether 64 patients from six centers in Germany were recruited (Table S1). After centralized pathological review, 48 patients were identified with histopathological diagnosis of adenocarcinoma of the colorectum and sufficient primary tumor material for further evaluations. Metastatic tissue obtained after first-line chemotherapy was available for NGS from 30 patients. Liquid biopsies taken before study enrollment were available from 42 patients. Liquid biopsies taken during and after first-line treatment were available from 44 patients. The study profile including sample numbers is depicted in Figure 1. Baseline and treatment characteristics of the patients are summarized in Table 1. The median duration of follow-up was 23.3 months.

### 3.2. Sequential Mutation Screening

Pre-therapeutic/primary tumor patient samples (P) from 48 out of 64 patients (75%) and 30 post-therapeutic/liver metastasis samples (M, 47%) were available for NGS analyses (Figure 1B, Tables S2 and S4). The analyses were successfully performed in 87.4% and 93.3% of P and M samples, respectively. In addition, the potential of a less-invasive approach, namely liquid (blood) biopsies, for molecular pathological characterization of untreated mCRCs, as well as their ability for longitudinal mutation monitoring were investigated. Therefore, 86 liquid biopsies, including 42 pre- (pre-L) and 44 intra-/post-therapeutic (i/p-L) liquid biopsies from 45 patients (70%) were successfully analyzed (100%) in a high-sensitivity setting (avg. coverage of all samples 16,553, avg. uniformity 91.6%). Full sample sets (P+pre-L+i/p L+M) were obtained from eight sets with all but M (P+pre-L+i/p L) from fourteen and sets missing only P (pre L+i/p L+M) from six patients. Moreover, sequencing data from six matched P and M as well as several single samples were analyzed. A detailed summary of the NGS results of all patient samples is visualized in Table S4. A typical mutational pattern of the colorectal carcinogenesis cascade [37] genes TP53 (mutation frequency: 69%), APC (57.1%), and KRAS (40.5%) was observed in P samples (Figure S1A). In addition, cancer-related mutations were found in P in 18 genes, including SMAD4 (11.9%), PIK3CA (11.9%), FBXW7 (9.5%), PTEN (7.1%), and BRAF (7.1%). These mutation frequencies are comparable to the ones found in the TCGA cohort. Similar mutation frequencies were detected in unmatched M tissue samples at 75.9% (TP53), 58.6% (APC), and 41.4% (KRAS). Co-mutations of TP53, APC, or KRAS were found in 57% of all cases and TP53+APC mutations showed the highest incidence (29.2%) (Figure S1B). Simultaneous mutations in all three genes were determined in 15.4%. Additional co-mutations to either one or more mutations in the three genes were detected in 22 genes, of which SMAD4 (13.8%), PIK3CA (13.8%), FBXW7 (10.8%), PTEN (7.7%), ATM (6.2%), and BRAF (6.2%) had the highest occurrence (Figure S1C).

**Figure 2:**
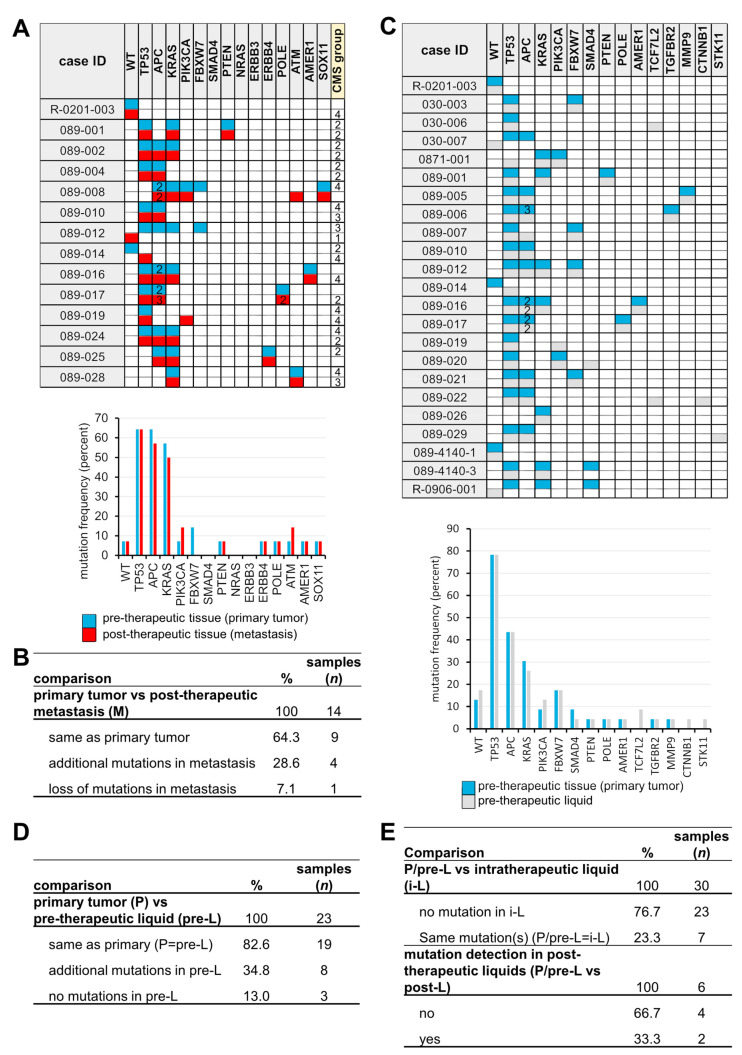
Pre-, intra-, and post-therapeutic mutational screening. (**A**) Mutations and frequencies (%) in matched P and M (*n* = 14). (**B**) Comparison of P vs. M. (**C**) Mutations and frequencies in matched P and pre-L (*n* = 30). (**D**) Comparison of P vs. pre-L. (**E**) Comparison of P/pre-L vs. i-L and P/pre-L vs. post-L.

In matched P and M samples (*n* = 14), the same mutations were found in nine cases (71.4%), whereas in four cases (28.6%), the identical mutational pattern as well as novel mutations were found (ATM, case 089-008; TP53, 089-014; 3rd TP53 and 2nd POLE, 089-017; PIK3CA, 089-019) (Figure 1A,B). In one case, none of the mutations present in P were detected in M. Since the PIK3CA mutation (089-019) was also present in the initial liquid biopsy at a low AF (3.9%) (Figure 2C), it probably does not reflect a resistance mechanism.

The same mutation spectra were found in matched P and pre-L samples in 19 out of 23 (82.6%) patients confirming that at time points close to P resection, sufficient amounts of ctDNA (circulating tumor DNA) were present in the blood to be detected by our high-sensitivity sequencing approach (Figure 2C,D). The positive predictive value (PPV) and negative predictive value (NPV) for KRAS mutation screening in pre-L were 1.0 and 0.94, respectively. Additional mutations were found in eight cases (34.8%), probably due to the higher sensitivity screening and lower AF limit (1% vs. 5%). In three patients (13%), no mutations were detected.

In follow-up liquid biopsies (i-L), pre-treatment mutations were found only in 7 out of 30 matched samples (P/pre-L vs. i-L) (23.3%), which can be interpreted as positive therapeutic response and/or lack of detectable ctDNA (Figure 2E, Table S4). Additional post-therapeutic liquids (p-L) were available from six patients. In two cases, a TP53 (AF 9.25%) and a KRAS (AF 2%) mutation were detected (Figure S1D,E), respectively, one to two months before metastasis resection in p-L (Figure 2E and Table S2), indicating the monitory capability of liquid biopsies.

In summary, we observed typical mutational patterns in the REVEAL patient cohort and confirmed the potential of liquid biopsy NGS analyses for mutation typing (e.g., RAS status) and for monitoring tumor progression or therapeutic response. However, mutational screening based on the customized 100-gene panel did not uncover any novel resistance mechanism.

### 3.3. Identification of a Post-Therapeutic Liver Metastasis CRC Expression Signature

**Figure 3:**
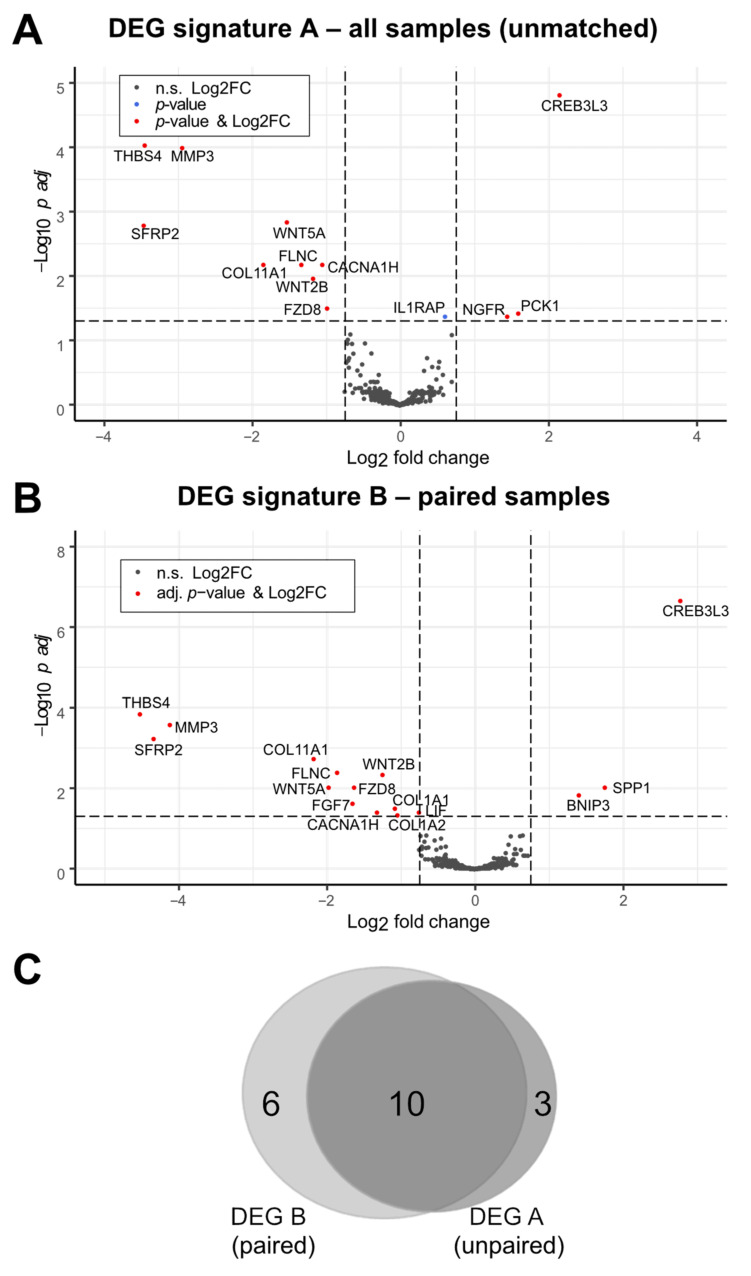
Identification of a post-therapeutic liver metastasis CRC expression signature. (**A**,**B**) Expression comparison in P and M tumor tissues in unmatched/all samples ((**A**) P, *n* = 29; M, *n* = 22) and paired samples ((**B**) P, *n* = 12; M, *n* = 12). Data were generated by moderated *t*-test with limma (padj, padjusted) and displayed by volcano plots. Differentially expressed genes (DEGs) are indicated by red dots. FC, fold change; n.s., not significant. p_adj_, p adjusted. (**C**) Venn diagram indicating the number of overlapping genes in DEG signatures A (unmatched) and B (paired).

In order to identify novel potential post-therapeutic biomarkers and mechanisms distinctly regulated by therapy in CRCs, we performed a comparative multiplex expression analysis utilizing pre-therapeutic primary tumor (P), pre-therapeutic normal tumor adjacent (N), and post-therapeutic liver metastasis (M) tissue. The mRNA expression of 770 cancer-related genes was measured in available tissues (P, *n* = 31; N, *n* = 31; M, *n* = 23), including matched P and M samples of 12 patients. After normalization, quality control, and data filtering, 26 N, 29 P, 22 M samples (Table S3), and 443 genes were chosen for further comparative analyses (Figure 1 and Figure S2A,B). Subsequent GSEA of P vs. M showed enrichment of the Hallmark_Epithelial_Mesenchymal_Transition gene set (N (*n* = 26) vs. P (*n* = 29); false discovery rate (FDR), 0.21; nominal p-value (nom-P), 0.11; and of the Hallmark_KRAS_Signaling_up gene set (P KRAS wildtype (WT) (*n* = 17) vs. P KRAS mutated (mut) (*n* = 8); FDR = 0.36; nom-P, 0.04) (Figure S2C,D). In addition, a normal CMS (consensus molecular subtype) group distribution was observed in P (*n* = 29; CMS1, 10.4%; CMS2, 37.9%; CMS3, 13.8%; CMS4, 37.9%) (Figure 2A and Figure S2E; Table S4). These results demonstrated the plausibility of the REVEAL patient cohort and dataset. Next, we aimed to identify differentially expressed genes (DEGs) in P vs. M in unmatched (P, *n* = 29; M, n = 22) and paired (from the same patient: P and M, *n* = 12) samples. Thirteen DEGs were identified in the unmatched (DEG signature A (DEG-A)) and sixteen DEGs in the paired (DEG signature B (DEG-B)) analyses (Table 2, Figure 3A,B). Ten DEGs were common in both signatures, whereas three and six DEGs were exclusive to DEG-A or DEG-B, respectively (Figure 3C). The majority of the DEGs were downregulated in the post-therapeutic metastatic setting (DEG-A 9/13; DEG-B 13/16). In both signatures, the strongest significant downmodulation (-) was observed for SFRP2 and THBS4, whereas the greatest expression increase (+) was found for CREB3L3. Seventeen out of the nineteen DEGs identified in both analyses were associated with 1) EMT/MET/Wnt regulation/signaling (SFRP2 (−), WNT5A (−), WNT2B (−), FZD8 (−), SPP1/OPN (+)), 2) extracellular matrix (ECM) modulation (MMP3 (−), COL11A1 (-), FLNC (−), FGF7 (−), COL1A1 (−), COL1A2 (−)), 3) endoplasmic reticulum (ER) stress/apoptosis (THBS4 (−), CACNA1H (−), BNIP3 (+), CREB3L3 (+)), and 4) NOTCH signaling/metabolism (PCK1 (+), LIF (−)). Moreover, IL1RAP, related to oncogenic signaling and NGFR, displaying an ambivalent role in tumor progression, were upregulated in M.

### 3.4. Association of the Expression Profile with Cellular Programs and Pathways

**Figure 4:**
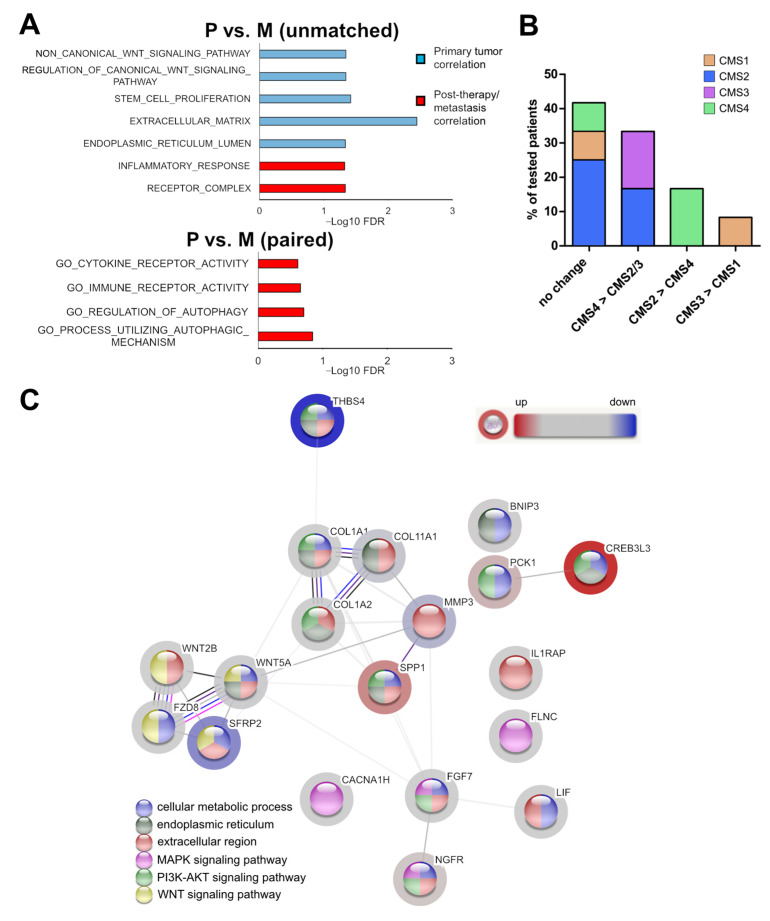
Association of the post-therapeutic liver metastasis CRC expression signature with cellular programs and pathways. (**A**) Gene ontology (GO) analyses of unmatched and paired samples utilizing all 443 gene expressions. (**B**) Changes in CMS classification from P to M in paired samples (*n* = 12). (**C**) STRING analysis based on the fold change of the 19 identified signature genes in M. Known and predicted interactions as well as examples of significantly enriched cancer related pathways are shown.

To further elucidate the biological differences in primary tumor and post-therapeutic metastatic specimens, we performed gene ontology (GO) analysis, GSEA, and CMS classification utilizing the complete 443 gene expression data. When comparing P vs. M (all unmatched samples), typical CRC gene sets such as EMT/Wnt/stem cell/NOTCH-related were significantly enriched in P (Figure S3). In contrast, EMT/Wnt (FDR = 0.0452) and stem cell proliferation (FDR = 0.0382) displayed negative associations in M (Figure 4A). Moreover, a negative correlation of the ECM (FDR = 0.00357) and positive enrichment of the inflammatory response (FDR = 0.0474), receptor complex (FDR = 0.0461), and ER lumen (FDR = 0.0461) gene sets was observed. Analyses of the paired sample expression sets revealed enrichment of the immune receptor activity (FDR = 0.221) and cytokine receptor activity (FDR = 0.242) as well as of two autophagy associated (FDR = 0.143 and 0.197) gene sets in M (Figure 4A). 

A similar trend was also determined in the unmatched setting (Figure S3). Consistent with EMT/ECM downmodulation, in 4 (33.3%) of the 12 paired P+M samples, a change from CMS4 (‘mesenchymal’) in P to CMS2 (‘canonical’) or CMS3 (‘metabolic’) (2 patients each) in M was observed. However, in two patients (16.7%), a switch from CMS2 (P) to CMS4 (M) was found (Figure 4B). A change from CMS3 to CMS1 (‘MSI, immune’) was noticed in one patient (8.3%), whereas no change in CMS classification was observed in five (41.7%) patients. In addition, a STRING (Search Tool for the Retrieval of Interacting Genes/Proteins) investigation based on the fold change of the 19 signature genes revealed amongst others (Table S5) a significant enrichment of the interaction datasets related to cellular metabolic process (12 genes, FDR = 0.00013), endoplasmic reticulum (8 genes, FDR = 0.0016), extracellular region (13 genes, FDR = 2.26 × 10^−6^), MAPK signaling pathway (4 genes, FDR = 0.0009), PI3K/AKT signaling pathway (8 genes, FDR = 2.73 × 10^−8^) and WNT signaling pathway (4 genes, FDR = 0.00011) (Figure 4C). Furthermore, we found 43 GO sets, including at least 10 out of the 19 signature genes significantly enriched within the large biological process section. Ten of the sets were related to response to stimuli/stress, and eight of them were associated with metabolic processes (Table S5). These results further support a downmodulation of EMT/Wnt signaling and ECM regulation in post-therapeutic metastatic CRCs. Moreover, immune and autophagy related mechanisms and metabolic processes seemed to be altered in this setting.

### 3.5. The Expression Pattern of the Post-Therapeutic Signature Genes Classifies Primary Tumor and Liver Metastasis of CRCs

**Figure 5:**
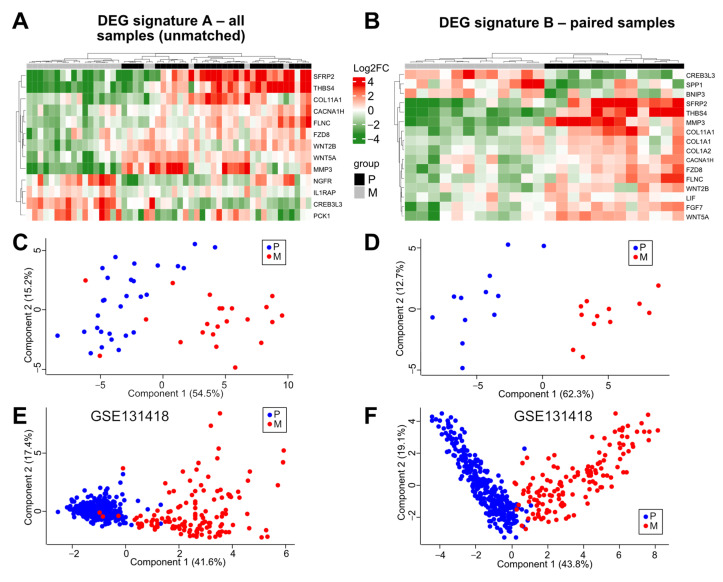
The post-therapeutic signature genes classifies primary tumor and liver metastasis. Comparison of P and M tumor tissue using DEG signature A (unmatched/all samples; (**A**,**C**)) and B (paired samples; (**B**,**D**)). (**A**,**B**) unsupervised heatmaps. Clustering of P and M samples is indicated. (**C**–**F**) Principal component analyses (PCA) utilizing genes included in DEG signature A (**C**,**E**) and B (**D**,**F**). (**C**,**D**) REVEAL data set. (**E**,**F**) GSE131418 data set.

After identification of the post-therapeutic signature genes and associated cellular programs, we investigated whether the expression pattern of these genes can correctly distinguish primary tumors and progression/metastatic samples of CRCs. As expected, we obtained a perfect separation by unsupervised hierarchical clustering and principal component analysis (PCA) of the paired samples when using the paired signature DEG-B (Figure 5B,D). By clustering of all samples utilizing the unmatched DEG-A genes, a perfect classification of the primary tumors (positive predictive value (PPV) = 1) was observed, whereas five metastases were wrongly classified (negative predictive value (NPV) = 0.85) (Figure 5A,C and Figure S5E). Application of the paired DEG-B signature to all samples resulted in a PPV of 0.81 and a NPV of 0.91 (Figure S4B,D,E). To confirm the capability of the REVEAL signature genes in classifying primary tumors and metastatic colorectal tissue, we performed PCAs with two independent datasets (GSE131418: P, n = 333; M (liver), *n* = 137 and GSE81582: P, *n* = 23; M, *n* = 19) with the DEG-A and B genes (Figure 4E,F and Figure S4F,G). Application of both signatures resulted in a clear separation of the primary tumors and metastatic samples. However, a better prediction (GSE131418) was obtained with DEG-A (area under the curve (AUC) = 0.964) compared with DEG-B (AUC = 0.693). These results indicate that the expression pattern of the DEG-A and B genes can classify primary tumors and (post-therapeutic) metastatic colorectal tissue.

### 3.6. Markers for the Sidedness of CRCs

**Figure 6:**
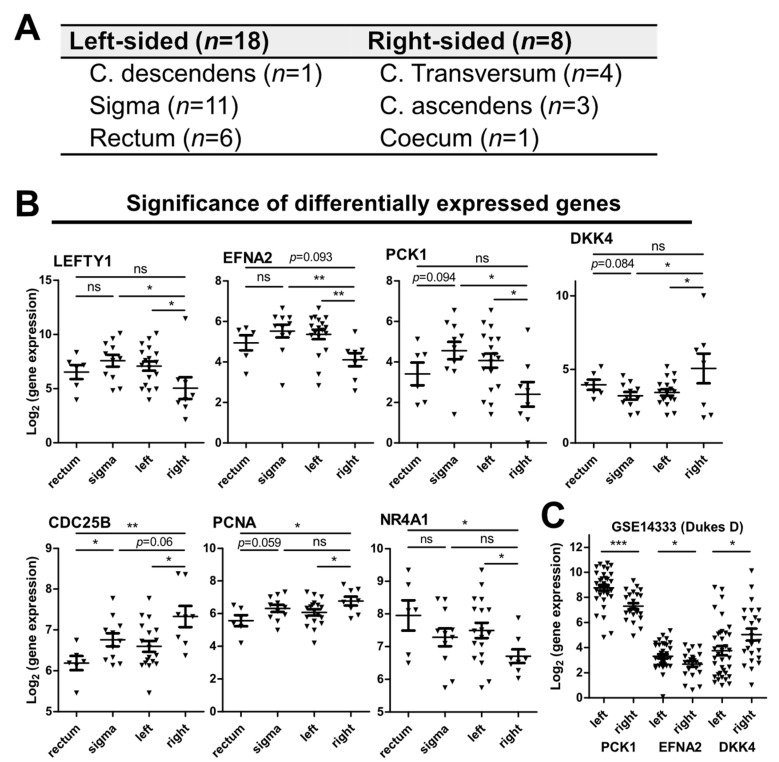
(**A**) Primary tumor localization of samples available for CRC sidedness association analyses. (**B**) Differentially expressed genes (DEGs) in primary CRCs of left- or right-sided origin. (**C**) Confirmation of side association of PCK1, EFNA2, and DKK4 expression in the independent data set GSE14333 (only Dukes’ D stage). (**B**,**C**) Significance levels were calculated by moderated *t*-test with limma. *, *p* < 0.05; **, *p* < 0.01; ***, *p* < 0.001; ns, not significant.

Patients with CRCs originating on the right side of the colon have a worse prognosis than patients with left-sided CRCs [38,39]. To find sidedness markers in the REVEAL cohort the expression pattern in left-sided primary tumors (L, *n* = 18) was compared with right-sided cancers (R, *n* = 8) (Figure 6). Seven genes displayed a significantly differential expression including the left–right asymmetry determination marker LEFTY1 [40]. The expression pattern of three additional genes was confirmed in an independent dataset GSE14333 (Dukes’ D; L, *n* = 36; R, *n* = 23) (Figure 6C). Namely, expression of EFNA2 (*p* = 0.0039) and, interestingly, of one of our signature genes PCK1 (*p* = 0.0117) were associated with left whereas DKK4 (*p* = 0.0235) with right-sidedness. This association was also observed in only sigmoid colon-derived cancers (*n* = 11) but not in cancers of rectal origin (*n* = 6) in the REVEAL cohort, supporting distinct molecular characteristics of sigmoid colon and rectal cancer [41].

### 3.7. Identification of Potential Biomarkers for Post-Therapeutic Metastatic CRCs

**Figure 7:**
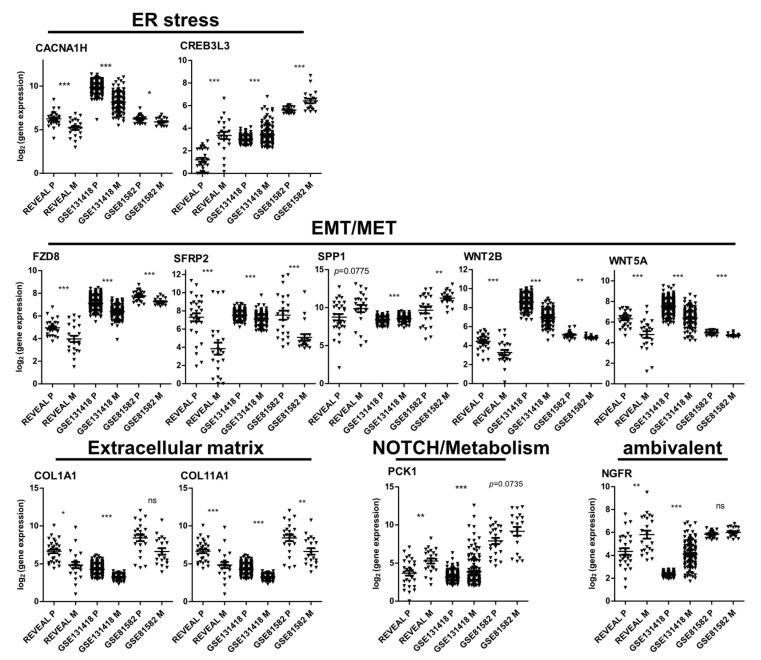
Expression of signature genes that showed the same significant trend (up/down regulation in M) in the REVEAL, GSE131418 and GSE81582 data sets. Associated cellular programs/pathways for each gene are indicated. Significance levels were calculated by moderated *t*-test with limma. *, p_adj_ < 0.05; **, p_adj_ < 0.01; ***, p_adj_ < 0.001; ns, not significant.

To search for genes with potential biomarker function, we initially analyzed the expression trend of the signature genes in two independent CRC gene expression datasets containing expression analyses of primary tumors and metastases (Figure 7). The expression pattern determined in our patient cohort was confirmed in the datasets GSE131418 and GSE81582 in 11 out of 19 genes, namely genes involved in (1) EMT/MET/Wnt FZD8, SFRP2, SPP1, WNT2B, WNT5A, (2) ECM modulation COL11A1, COL1A1, (3) ER-stress CACNA1H, CREB3L3, (4) NOTCH/metabolism PCK1 and NGFR (Figure 7, Table S6). Moreover, expression of the majority of the genes (CACNA1H, COL1A1, COL11A1, FZD8, NGFR, SFRP2, WNT2B, and WNT5A) was associated with the ‘mesenchymal’ CMS group 4 in the TCGA (The Cancer Genome Atlas) Colorectal Adenocarcinoma dataset [42] (Figure S5). SPP1 was correlated with CMS1 and CMS4, whereas no clear association was observed for PCK1 and CREB3L3. All of the CMS4-associated genes except NGFR and SPP1 were downregulated in the metastases of the REVEAL cohort and functionally associated with EMT/WNT or ECM modulation, further supporting a reversion of the mesenchymal/EMT phenotype. These findings confirm and further characterize the identified 11 signature genes and suggest that they may represent potential biomarkers for progressive/metastatic CRCs.

### 3.8. Clinical Association of the Identified Signature Genes

**Figure 8:**
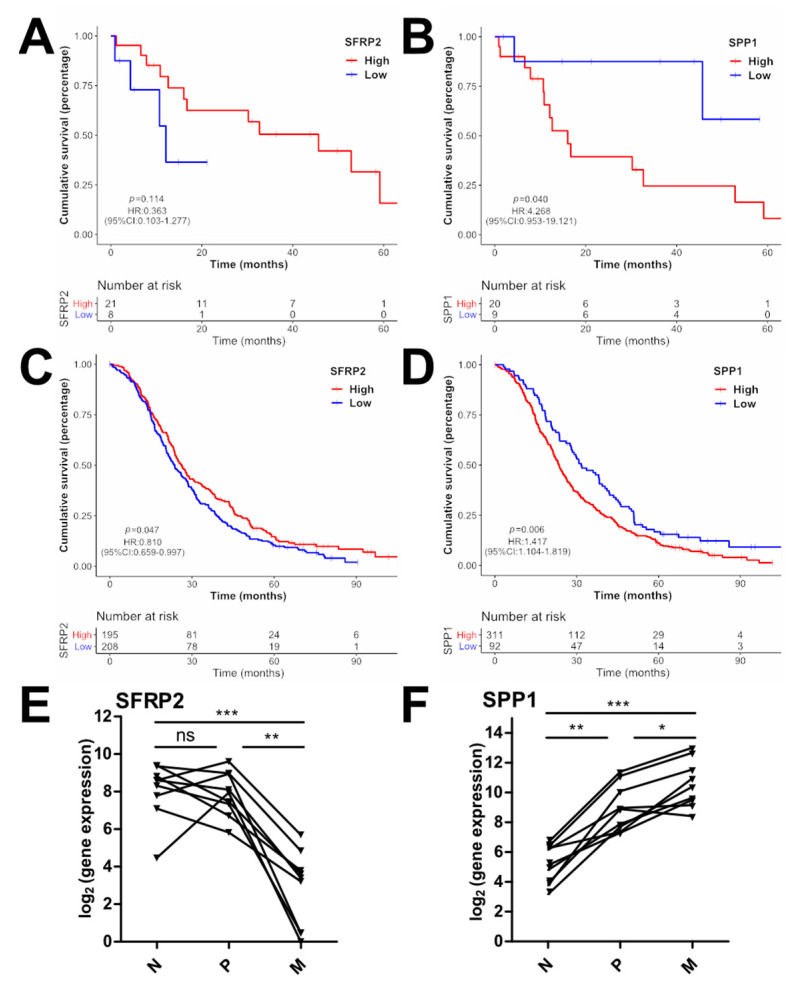
Prognostic role of the identified signature genes. (**A**–**D**) Overall survival (OS) of patients of the REVEAL (**A**,**B**) and FIRE-3 (**C**,**D**) cohorts expressing high (red curve) or low (blue curve) levels of SFRP2 (**A**,**C**) or SPP1 (**B**,**D**); *p* values were calculated by log-rank test. (**E**,**F**) Expression of SFRP2 and SPP1 in paired normal tissue (N), primary tumor (P), and metastasis (M) (*n* = 9). *, *p* < 0.05; **, *p* < 0.01; ***, *p* < 0.001, ns, not significant.

Next, we investigated the clinical importance and potential prognostic association by survival analyses in relation to the expression levels of the signature genes in primary tumors. An impact on overall survival was estimated only for SFRP2 (secreted frizzled-related protein 2) and SPP1/OPN (secreted phosphoprotein 1/osteopontin) with SFRP2_low_ (low, *n* = 8, OS = 12.62m; high, *n* = 21, OS = 36.47m; HR = 0.363; 95% CI = 0.103–1.277; *p* = 0.114) and SPP1_high_ (low, *n* = 9, OS = 47.86m; high *n* = 20, OS = 25.14; HR = 4.268; 95% CI = 0.953–19.121; *p* = 0.040) (Figure 8A,B). To validate the survival in a larger patient cohort, the prognostic relevance of the identified 11 signature genes was analyzed in the FIRE-3 trial [43] expression dataset (ALMAC’s Xcel™ gene-expression array, *n* = 403). This trial investigated standard first line chemotherapy with FOLFIRI in conjunction with either cetuximab or bevacizumab. Significant overall survival associations were observed for SFRP2_low_ (low, *n* = 208, OS = 23.66m; high, *n* = 195, OS = 26.25m; HR = 0.81; 95% CI = 0.659–0.997; *p* = 0.047) and SPP1_high_ (low, *n* = 92, OS = 31.67; high, *n* = 311, OS = 23.62m; HR = 1.417; 95% CI = 1.104–1.819; *p* = 0.006) (Figure 8C,D).

Since high SPP1 expression in primary CRC tumors was previously associated with poor prognosis [44] and SFRP2 promoter methylation resulting in reduced SFRP2 expression was considered a CRC biomarker [45], the expression during the course of cancer progression was analyzed in paired normal (N), P and M samples of the REVEAL study (*n* = 9) (Figure 8E,F). A continuous increase in SPP1 from N to P (*p* < 0.01) to M (*p* < 0.05) was observed in seven patients (77.77%), whereas a decrease from N to P (n.s.) to M (*p* < 0.01) was measured for SFRP2 in six patients (66.66%). These results further support the roles of SFRP2 and SPP1 as CRC biomarkers.

## 4. Discussion

Here we report results from the prospective observational biomarker study REVEAL (ReEVAluation of Liver metastasis). This study aimed to identify molecular alterations and resistance mechanisms acquired during standard first-line treatment against mCRC in primary tumor and post-treatment metastatic tissues. Furthermore, we used circulating tumor DNA (ctDNA) prepared from liquid biopsies to monitor treatment response and emerging alterations conferring resistance against systemic treatment.

Analyses of genetic alterations in primary tumor samples before treatment (P) revealed a typical mCRC mutational pattern as previously observed in other studies [8,9,20]. Comparative analyses of matched P and tumor specimens from liver metastases after standard first-line treatment (M) showed the same mutations in 71.4% of the patients, whereas in 28.6%, metastasis private mutations were found in M. Of note, these additional mutations in the *ATM*, *TP53*, *POLE* genes do not reflect typical resistance mutations in solid tumors. Other studies described a similar mutational pattern in primary tumors and metastases of colorectal origin as well [8,9]. Notably, most of these studies compared untreated primary tumors and metastases. Nevertheless, our study confirms that standard treatment does not mainly change the metastatic genome.

By utilization of a high sensitivity massive parallel sequencing (MPS) approach, we also investigated pre-, intra-, and post-therapeutic liquid biopsies (L). The same mutation spectra were observed in 83% of matched P and pre-L samples, reflecting the results of another study in mCRC [19]. This confirms the feasibility of liquid biopsy screening with high-sensitivity MPS analyses. In addition, our results support the still-understudied value of blood-based RAS typing for guiding anti-EGFR therapy in metastatic CRC patients [16,19]. The PPV (1.0) and NPV (0.94) for *KRAS* mutations utilizing our approach were comparable to the values reported by Schmiegel et al. [19,46]. In addition, we measured therapeutic responses in intra- and post-therapeutic liquid biopsies as indicated by lack of mutation detection in those samples. However, this may also be mediated by decreased shedding of genomic DNA fragments from tumor cells during treatment and inadequate sensitivity of the used approach: all well-known limitations of ctDNA monitoring [46].

We could not determine novel candidate resistance mechanisms to standard treatment of mCRC based on mutational analysis. Therefore, we subsequently analyzed transcriptional changes in 770 cancer-associated genes in the remaining primary tumor and metastatic tissue samples with sufficient material for RNA analyses. The analyses of all available and matched pair samples revealed, in total, 19 significantly differentially expressed genes (DEGs) between P and M. The DEGs are known to be associated with extracellular matrix (ECM) modulation, epithelial–mesenchymal transition (EMT)/mesenchymal–epithelial transition (MET) regulation, endoplasmic reticulum (ER) stress, and metabolism regulation, as well as oncogenic signaling via MAPK-, NOTCH-, PI3K/AKT- and WNT-pathways. We observed a negative enrichment of the EMT gene set and altered expression of five DEGs involved in EMT/Wnt signaling in M, corroborating previous findings of EMT reversion in metastases [10,47]. This was further supported by the downmodulation of ECM-regulating factors in the REVEAL cohort and other studies [47]. Moreover, we observed a positive correlation of immune/cytokine receptor, inflammatory response, and autophagy gene sets in the M group. In the past decade, the involvement of the microenvironment and especially the role of the immune system and inflammation triggering aggressiveness, metastasis, and therapy response of cancer cells have been highlighted [48,49,50]. Recent metastases expression profiling studies utilized multiple design approaches and further underlined the importance of immune signatures and even further differentiation of metastatic subtypes. Comparison of metastatic and non-metastatic primary tumors established a 115 gene metastatic expression signature and emphasized the importance of Wnt and TGFβ signaling [51]. Another study identified several coding and non-coding genes differentially expressed in primary tumors and cytotoxic therapy-naïve metastases associated with endocytosis, cell cycle, PI3K/AKT, and TGFβ signaling [11]. Kamal et al. identified two mCRC metastasis subtypes characterized by an EMT/inflammatory and a proliferative signature in a comprehensive analysis of two large patient cohorts [10]. Currently, the prognostic and therapeutic value of immune signatures for various tumors is being investigated in clinical trials. Our results further support the need for diagnostic surveillance of tumor samples during the course of systemic treatment for changes in the immune/inflammatory response to potentially support clinical decision making.

Recently, multiple studies addressed the prognostic impact of consensus molecular subtypes (CMS) and their predictive effects on different combinatorial treatments of mCRCs [43,52,53]. In contrast to the clinical association of CMS groups previously reported in early-stage cohorts [54] in mCRCs, CMS2 and CMS3 predicted best overall survival [43,52,53]. The REVEAL patient cohort showed a similar CMS group distribution in primary tumors compared with other studies [54]. Interestingly, a switch to a different CMS group was observed from P to M in 58.3% of the matched samples. Molecular subtype shifting was also reported in metastases [10] and in the budding/EMT region of primary tumors [55]. Additionally, it is well-known that chemotherapy and targeted approaches significantly affect gene expression and thus the molecular classification of CRCs [10,49,56]. This suggests that CMS groups are adaptable according to the stage of the tumor cells (primary tumor/EMT/MET/metastasis) and are influenced by standard treatment. Thus, a re-evaluation of CMS groups during/after treatment may be taken into consideration in future clinical trials.

We evaluated the clinical relevance of identified DEGs between P and M by correlating gene expression with patient survival. Within the REVEAL cohort, a trend was observed for *SFRP2* (secreted frizzled-related protein 2) and a significant correlation for *SPP1*/*OPN* (secreted phosphoprotein 1/osteopontin). Due to the restricted number of patients, we expanded the survival analyses to the larger FIRE-3 patient cohort. The FIRE-3 study compared standard first-line chemotherapy with either cetuximab or bevacizumab, as previously described [4]. In agreement with the second-strongest downregulation of *SFRP2* in metastases in the REVEAL trial, low *SFRP2* expression was significantly correlated with a reduced OS. SFRP2 is a secreted key inhibitor of non-canonical Wnt signaling and is considered a tumor suppressor gene depending on the organ/cellular context [57]. *SFRP2* expression is downregulated in cancer cells by promoter hypermethylation. Therefore, *SFRP2* methylation may also represent a promising biomarker for CRC in blood and stool samples [57]. Although *SFRP2* was shown to act as tumor suppressor in CRC cell lines and *SFRP2* methylation is a hallmark of CRC tumor cells, the clinical prognosis of *SFRP2* methylation is contradictory. Some studies estimated a poor and other studies a favorable clinical outcome in CRCs with hyper-methylated *SFRP2* [57]. This difference may be due to variable SFRP2 secretion from stroma cells [58]. We observed a continuous reduction from normal to primary tumor to metastatic tissues in the majority of our matched samples and a worse prognosis in patients of the FIRE 3 cohort with low *SFRP2* expression. These findings support the importance of *SFRP2* as a tumor-suppressing biomarker, the loss of which leads to poor prognosis. However, the influence of the tumor microenvironment should also be considered in future studies, as well as a predictive relevance regarding targeted therapy.

Moreover, higher *SPP1* expression was associated with poor survival. Similar observations were made in other trials based on investigations of primary tumors and blood plasma [44,59]. Interestingly, we determined a continuous upregulation of *SPP1* from normal tissue to primary tumors and ultimately to metastases, which helps to explain the association of *SPP1* plasma levels with post-operative metastasis [59]. *SPP1*/*OPN* encodes a secreted integrin- and CD44-binding factor that mediates PI3K/AKT, NF-κB, and MAPK signaling and thus drives CRC progression and chemoresistance [60]. Therefore, our study further emphasizes the role of *SPP1* not only as a prognostic factor that can be quantified from body fluids but also as a potentially druggable target, supporting the development of inhibitors targeting the SPP1/integrin axis.

The prospective multicentric REVEAL study has limitations. A restricted number of patient samples was available for mutational and gene expression analyses. Especially, the number of whole sets of matched samples was limited partly due to material restrictions (sufficient tissue for DNA and RNA preparation was required) and patient’s rejection of additional liver biopsies in case of progression during first-line treatment. To compensate for inequal recruitment, we analyzed matched as well as unmatched tumor samples and validated our results in two independent datasets and in the large phase III trial FIRE-3. However, the power of statistical analyses was limited and further sub-grouping by e.g., different treatment regimens or mutational subtypes was not possible.

## 5. Conclusions

The REVEAL study indicates that serial mutational and gene expression analysis is feasible and a promising approach to elucidate metastatic progression, treatment effects, and drug resistance. Consequently, future studies should examine larger cohorts of matched samples. Particularly, the analysis of metastases during and after first-line treatment, including CMS classification, might be a promising approach for guiding second-line therapies. Moreover, the detection of immune/inflammatory expression signatures and the potential of *SFRP2* as prognostic biomarker and *SPP1* as potentially druggable target warrants future studies with larger cohorts.

## Figures and Tables

**Table 1:** Patient and tumor baseline characteristics.

Characteristics	*N*	%
Age		
Age-median	62 (range 20–87)	
Sex		
Male	38	59.4
Female	26	40.6
Performance status		
ECOG 0–1	58	90.6
ECOG 2–3	2	3.1
NA	4	6.3
Primary tumor sidedness		
Right-sided	13	20.3
Left-sided	49	76.6
NA	2	3.1
T-stage of primary		
T1-2	13	20.3
T3-4	39	60.9
NA	12	18.8
N-stage of primary		
N0	7	10.9
N1	9	14.1
N2	11	17.2
NA	22	57.8
Grading of primary		
G1-2	44	68.8
G3	8	12.5
NA	12	18.8
Metastasis		
synchronous	48	75
metachronous	16	25
Number of metastatic sites		
1 site	25	39.1
≥2 sites	39	60.9
Chemotherapy		
FOLFOXIRI	6	9.4
plus Bevacizumab	3	4.7
plus Panitumumab	3	4.7
FOLFOX	22	34.4
plus Panitumumab	2	3.1
plus Bevacizumab	11	17.2
plus Cetuximab	2	3.1
FOLFIRI	21	32.8
plus Bevacizumab	7	10.9
plus Cetuximab	9	14.1
Capecitabine	4	6.3
plus Irinotecan & Bevacizumab	1	1.6
plus Bevacizumab	2	3.1
Cetuximab mono	1	1.6
RAS mutation		
no	37	57.8
yes	24	37.5
NA	3	4.7
BRAF mutation		
no	54	84.4
yes	3	4.7
NA	7	10.9
total	64	100

**Table 2:** Significant DEGs identified in all samples (DEG A) and paired samples (DEG B).

Gene	DEG	Log_2_ FC	Avg Expr	*p* Value	*P*_adj_	FC	%Change	Program/Pathway/Function
***SFRP2***	**A**	**−3.46**	**5.81**	**1.77 × 10^−5^**	**0.0016**	**0.09**	**−90.91**	**EMT/MET/WNT**
	**B**	**−4.33**	**4.94**	**5.85 × 10^−6^**	**0.00065**	**0.05**	**−95.03**	
***THBS4***	**A**	**−3.44**	**4.55**	**3.98 × 10^−7^**	**8.81 × 10^−5^**	**0.09**	**−90.79**	**ER stress, tumor suppressor CRC**
	**B**	**−4.52**	**4.05**	**7.26 × 10^−7^**	**0.00016**	**0.04**	**−95.64**	
***MMP3***	**A**	**−2.94**	**4.24**	**6.56 × 10^−7^**	**9.69 × 10^−5^**	**0.13**	**−86.97**	**ECM modulating/related**
	**B**	**−4.11**	**3.76**	**1.99 × 10^−6^**	**0.00029**	**0.06**	**−94.21**	
***COL11A1***	**A**	**−1.84**	**5.89**	**0.00012**	**0.0065**	**0.28**	**−72.07**	**ECM modulating/related**
	**B**	**−2.17**	**5.73**	**2.21 × 10^−5^**	**0.002**	**0.22**	**−77.78**	
***WNT5A***	**A**	**−1.53**	**5.66**	**1.28 × 10^−5^**	**0.0014**	**0.35**	**−65.37**	**EMT/MET/WNT**
	**B**	**−1.97**	**5.41**	**0.00022**	**0.0098**	**0.26**	**−74.47**	
***FLNC***	**A**	**−1.33**	**5.90**	**9.01 × 10^−5^**	**0.0065**	**0.40**	**−60.22**	**ECM modulating/related**
	**B**	**−1.85**	**5.73**	**5.75 × 10^−5^**	**0.0042**	**0.28**	**−72.26**	
***WNT2B***	**A**	**−1.17**	**3.93**	**0.00022**	**0.011**	**0.44**	**−55.56**	**EMT/MET/WNT**
	**B**	**−1.24**	**3.61**	**7.2 × 10^−5^**	**0.0046**	**0.42**	**−57.66**	
***CACNA1H***	**A**	**−1.05**	**5.80**	**0.00011**	**0.0065**	**0.48**	**−51.70**	**ER stress, inh. of proliferation**
	**B**	**−1.32**	**5.66**	**0.0013**	**0.038**	**0.40**	**−59.95**	
***FZD8***	**A**	**−0.98**	**4.53**	**0.00071**	**0.031**	**0.51**	**−49.30**	**EMT/MET/WNT**
	**B**	**−1.62**	**4.30**	**0.00022**	**0.0098**	**0.33**	**−67.47**	
*FGF7*	B	−1.65	4.00	0.00067	0.025	0.32	−68.14	ECM modulating/related
*COL1A1*	B	−1.07	11.31	0.00093	0.032	0.48	−52.37	ECM modulating/related
*COL1A2*	B	−1.04	8.57	0.0017	0.047	0.49	−51.37	ECM modulating/related
*LIF*	B	−0.75	6.01	0.0013	0.038	0.59	−40.54	NOTCH inihibition
*IL1RAP*	A	0.61	4.78	0.0012	0.042	1.53	52.63	Oncogenic signaling
*BNIP3*	B	1.41	5.32	0.00038	0.015	2.66	165.74	ER stress, apoptosis, autophagy
*NGFR*	A	1.45	4.98	0.0012	0.042	2.73	173.21	Ambivalent, tumor suppressor CRC
*PCK1*	A	1.6	4.37	0.00092	0.037	3.03	203.14	NOTCH, metabolism
*SPP1/OPN*	B	1.76	9.70	0.00021	0.0098	3.39	238.70	EMT/MET/WNT
***CREB3L3***	**A**	**2.16**	**2.13**	**3.33 × 10^−8^**	**1.48 × 10^−5^**	**4.47**	**346.91**	**ER stress, transcription factor**
	**B**	**2.78**	**2.54**	**5.23 × 10^−10^**	**2.32 × 10^−7^**	**6.87**	**586.85**	

Avg expr, average expression (log_2_); ECM, extracellular matrix; EMT, epithelial to mesenchymal transition; ER, endoplasmic reticulum; FC, fold change; MET, mesenchymal to epithelial transition. Padj, adjusted *p* value. Genes found in DEG A and B are highlighted in bold.

## Data Availability

Normalized Nanostring expression data are shown in Figure S2. Sequencing data presented in this study are available on request from the corresponding author.

## Supplementary Materials

The following supporting information can be downloaded at: https://www.mdpi.com/article/10.3390/cancers14153631/s1, Figure S1: Mutation screening in the REVEAL cohort; Figure S2: Quality control and plausibility of the REVEAL expression data set; Figure S3: Gene ontology (GO) analyses; Figure S4: Principal component analyses and unsupervised hierarchical clustering; Figure S5; CMS group association of the confirmed signature genes in the TCGA Colorectal Adenocarcinoma data set; Table S1: Recruitment centers; Table S2: Summary of sequencing coverage and quality statistics for each sample; Table S3: Samples/RNAs used in Nanostring expression analyses and normalized expression data; Table S4: Summary of the NGS results of all available patient samples; Table S5: STRING analysis results; Table S6: Signature genes in the REVEAL cohort with similar expression trend in two other CRC data sets.
