# Supplementary material for: Serial Analysis of Gene Mutations and Gene Expression during First-Line Chemotherapy against Metastatic Colorectal Cancer: Identification of Potentially Actionable Targets within the Multicenter Prospective Biomarker Study REVEAL
Source: Cancers (Basel). 2022 Jul 26;14(15):3631. doi: 10.3390/cancers14153631 (PMC9367450; doi:10.3390/cancers14153631)
Supplement: Supplementary file 1 [file cancers-14-03631-s001.zip › Supplementary Materials Kumbrink et al..pdf]

Supplementary Material

Serial analysis of gene mutations and gene expression during first-line chemotherapy against metastatic colorectal cancer: Identification of potentially actionable targets within the multicenter prospective biomarker study REVEAL

Jörg Kumbrink [1,2], Lisa Bohlmann [1], Soulafa Mamlouk [3,4,17], Torben Redmer [5], Daniela Peilstöcker [1], Pan Li [1], Sylvie Lorenzen [6], Hana Algül [7,8], Stefan Kasper [9], Dirk Hempel [10,11], Florian Kaiser [12], Marlies Michl [13,14], Harald Bartsch [1], Jens Neumann [1,2], Frederick Klauschen [1,2], Michael von Bergwelt-Baildon [2,13], Dominik Paul Modest [3,15], Arndt Stahler [3,15], Sebastian Stintzing [3,4,15], Andreas Jung [1,2], Thomas Kirchner [1,2], Reinhold Schäfer [3,16], Volker Heinemann [2,13,14], Julian W. Holch [2,13,14]

[1] Institute of Pathology, Faculty of Medicine, Ludwig-Maximilians-University (LMU) Munich, Munich, Germany.
[2] German Cancer Consortium (DKTK), partner site Munich, Germany.
[3] German Cancer Consortium (DKTK), partner site Berlin, Germany.
[4] German Cancer Research Center (DKFZ), Heidelberg, Germany.
[5] University of Veterinary Medicine Vienna, Institute of Medical Biochemistry, Vienna, Austria.
[6] Klinikum rechts der Isar, Technical University of Munich, III. Medizinische Klinik und Poliklinik, Munich.
[7] Technical University of Munich, Germany, School of Medicine.
[8] Comprehensive Cancer Center Munich, Technical University of Munich, Klinikum rechts der Isar, Germany.
[9] Department of Medical Oncology, West German Cancer Center, University Hospital Essen, Essen, Germany.
[10] Steinbeishochschule Berlin, Berlin, Germany.
[11] Steinbeis Institut für klinische Hämatoonkologie, Donauwörth.
[12] VK&K Studien GbR, Landshut, Germany.
[13] Department of Medicine III, University Hospital, LMU Munich, Munich, Germany.
[14] Comprehensive Cancer Center, University Hospital, LMU Munich, Munich, Germany.
[15] Department of Hematology, Oncology and cancer Immunology (CCM) Charité – Universitätsmedizin Berlin. Germany.
[16] Charité Comprehensive Cancer Center, Charité Universitätsmedizin Berlin, Germany.
[17] Institute of Pathology, Charité Universitätsmedizin Berlin, Berlin, Germany.
* Correspondence: Joerg.Kumbrink@med.uni-muenchen.de

Figure S1. Mutation screening in the REVEAL cohort.

Figure S2. Quality control and plausibility of the REVEAL expression data set.

Figure S3. Gene ontology (GO) analyses.

Figure S4. Principal component analyses and unsupervised hierarchical clustering.

Figure S5. CMS group association of the confirmed signature genes in the TCGA Colorectal Adenocarcinoma data set.

Table S1. Recruitment centers.

Table S2 (excel file). Summary of sequencing coverage and quality statistics for each sample.

Table S3 (excel file). Samples/RNAs used in Nanostring expression analyses and normalized expression data.

Table S4 (excel file). Summary of the NGS results of all available patient samples.

Table S5 (excel file). STRING analysis results.

Table S6. Signature genes in the REVEAL cohort with similar expression trend in two other CRC data sets.

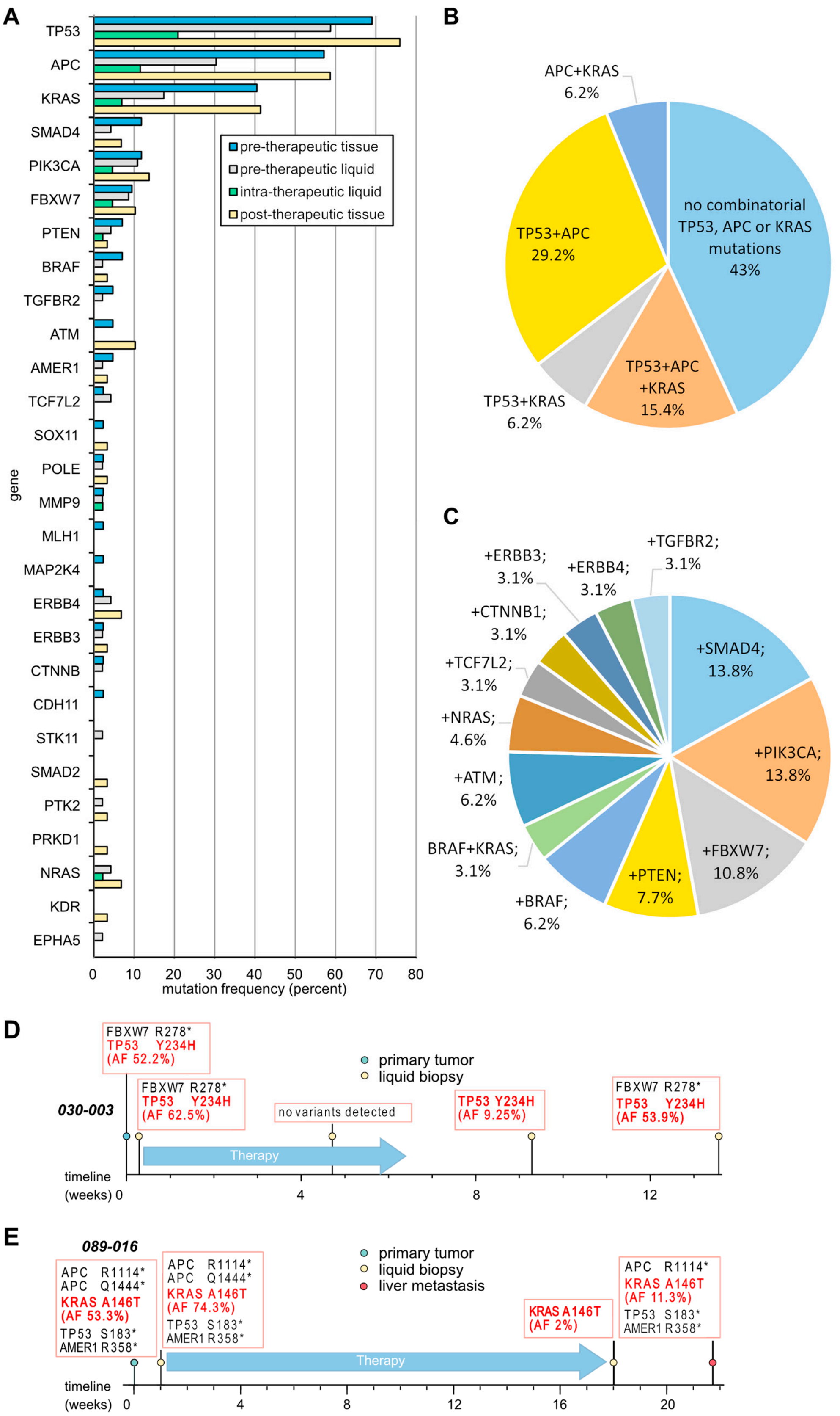

Figure S1. Mutation screening in the REVEAL cohort. (**A**) Mutation frequencies (%) in P (*n*=43), pre-L (*n*=42), i/p-L (*n*=44) and M (*n*=28). (**B**) Frequencies (% of all cases) of combinatorial mutations in the TP53, APC and/or KRAS genes. (**C**) Frequencies (%) of cases with either one or more mutations in TP53, APC, and/or KRAS and mutations in additional genes. (**D**,**E**), Examples of monitoring the mutational pattern in pre-, intra- and post-therapeutic tissue and/or liquid samples from single patients.

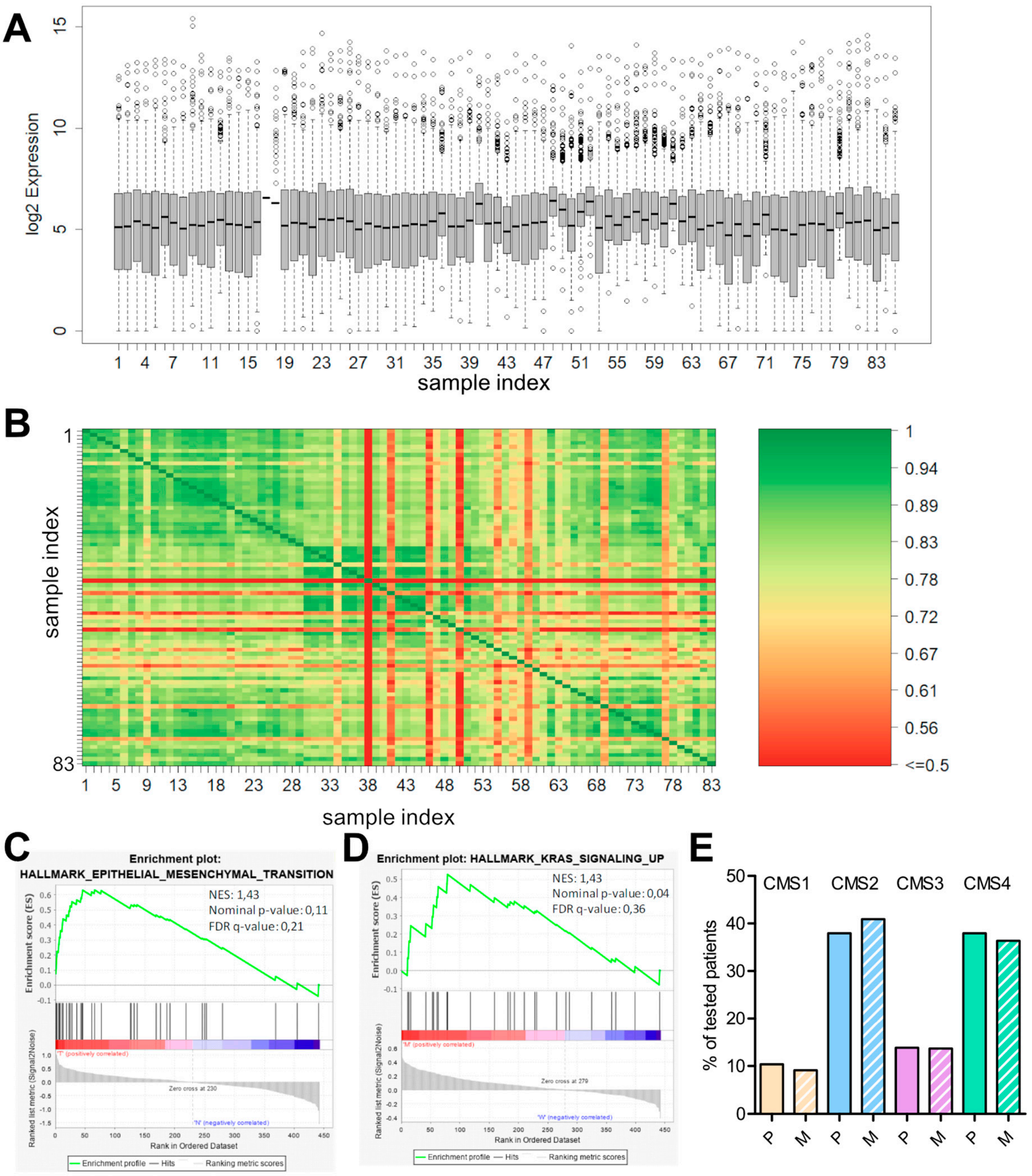

Figure S2. Quality control (**A**,**B**) and plausibility of the REVEAL expression data set (**C**,**D**). **A**, Box plot diagram displaying data after normalization with the nSolver algorithm. B, Similarity matrix (spearman correlation) of all samples. (**A**,**B**), Sample 17, 18, 40, 43, 48, 52 and 61 were removed from further analysis for not reaching our quality standards. (**C**), GSEA analysis comparing the expression data from primary tumors (T, *n*=29) to normal surrounding tissue (N, *n*=26). The primary CRC tumor expression pattern is associated with the EMT dataset confirming the plausibility of the data. (**D**), GSEA analysis comparing the expression data from KRAS mutated (M, *n*=8) to KRAS wildtypic primary tumors (W, *n*=17). The KRAS mutated primary tumor expression pattern is associated with the KRAS signaling dataset confirming the plausibility of the data. (**E**), CMS classification of primary tumors (P; *n*=29) and metastases (M; *n*=22). NES, normalized enrichment score. FDR, false discovery rate.

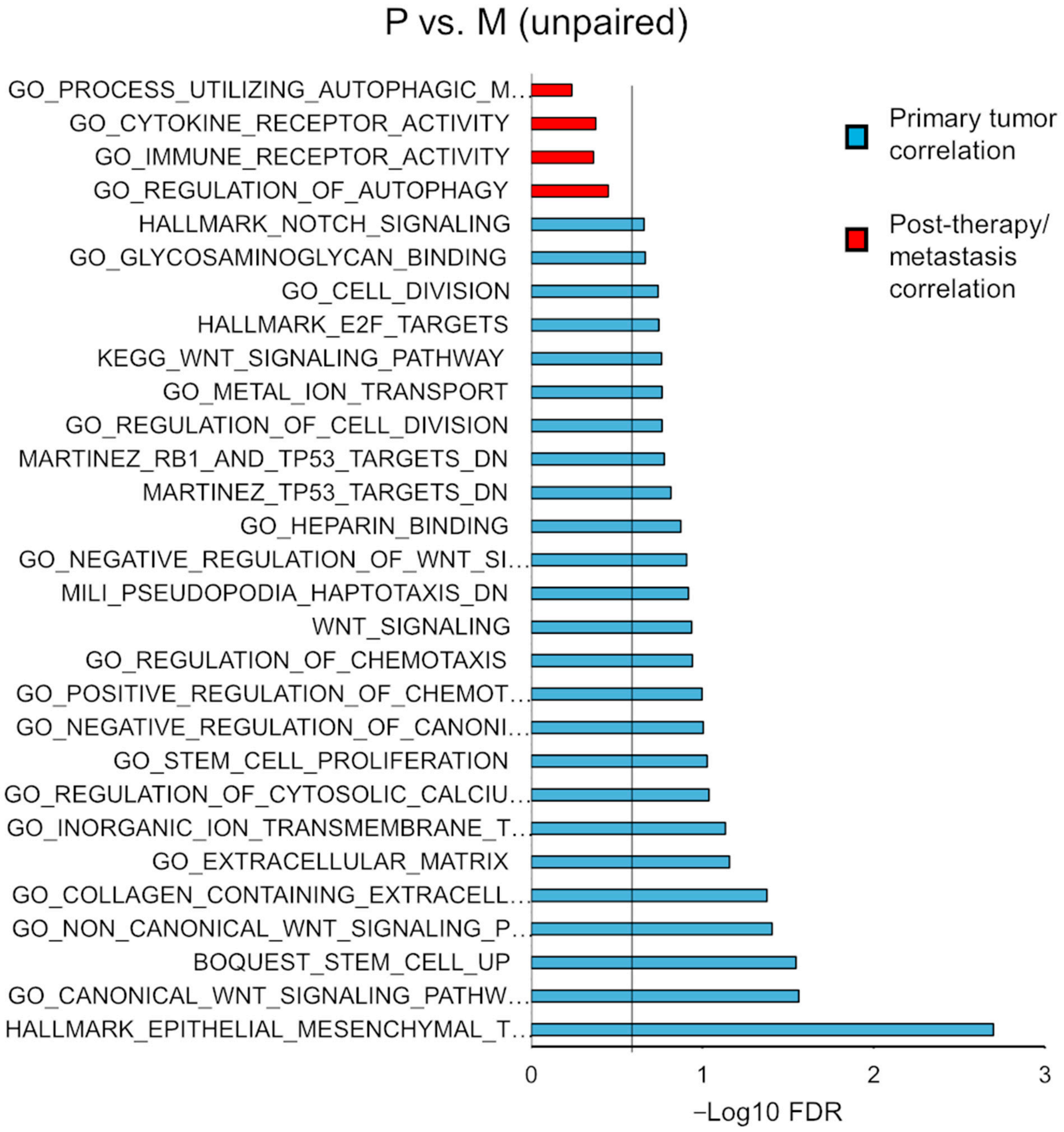

Figure S3. Gene ontology (GO) analyses of unmatched samples utilizing all 443 gene expressions. P or M correlations are indicated. FDR, false discovery rate.

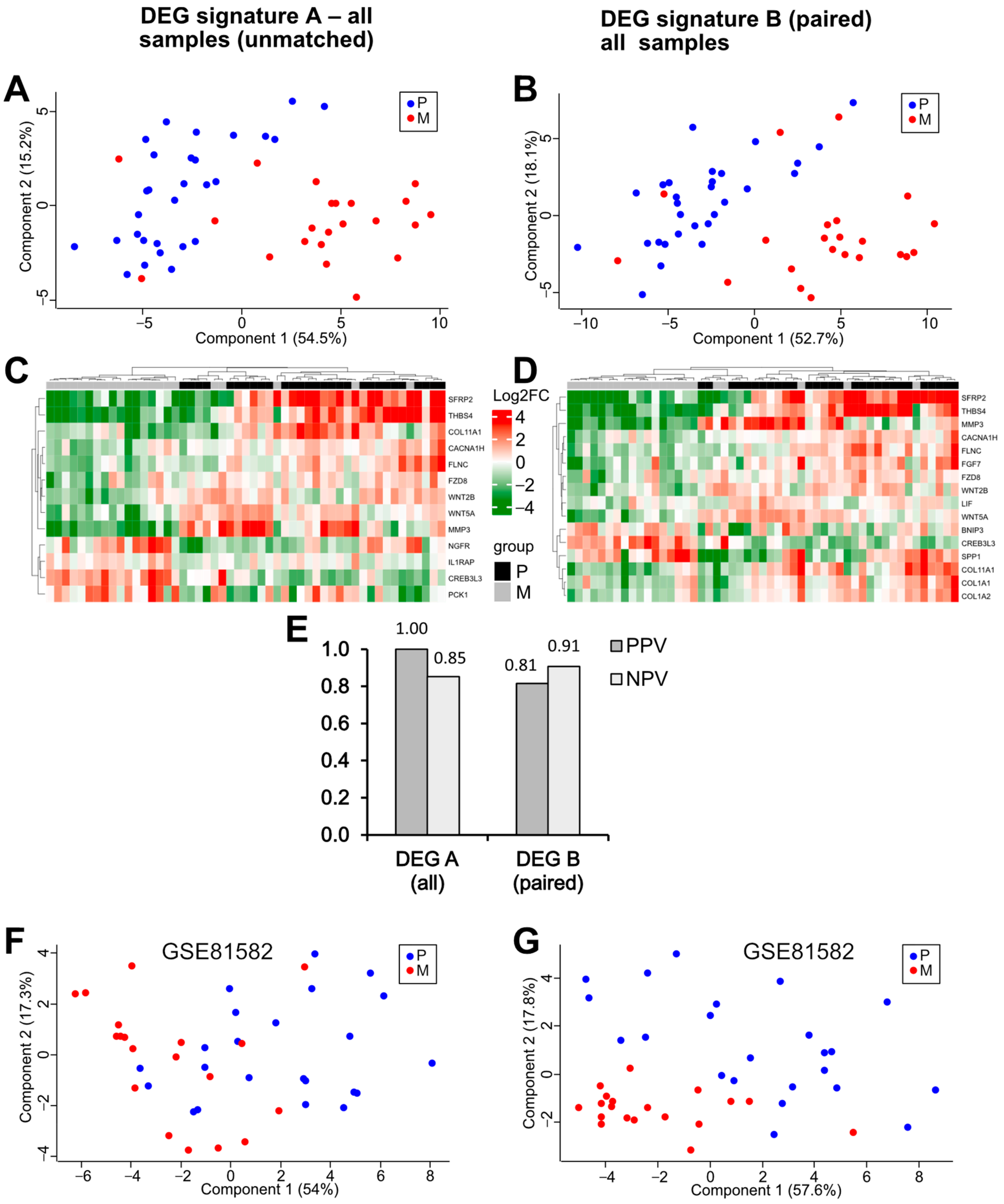

Figure S4. Principal component analyses (PCA) (**A**,**B**) and unsupervised hierarchical clustering (**C**,**D**) utilizing the REVEAL data set and DEG signature A (left) and B (right) applied to all samples (*n*=51). (**E**), Positive predictive (PPV) and negative predictive value (NPV) for classifying P and M of DEG A and B applied to all samples. (**F, G**), PCA utilizing data set GSE81582 (P, *n*=23; M, *n*=19) and genes included in DEG signature A (**F**) and B (**G**).

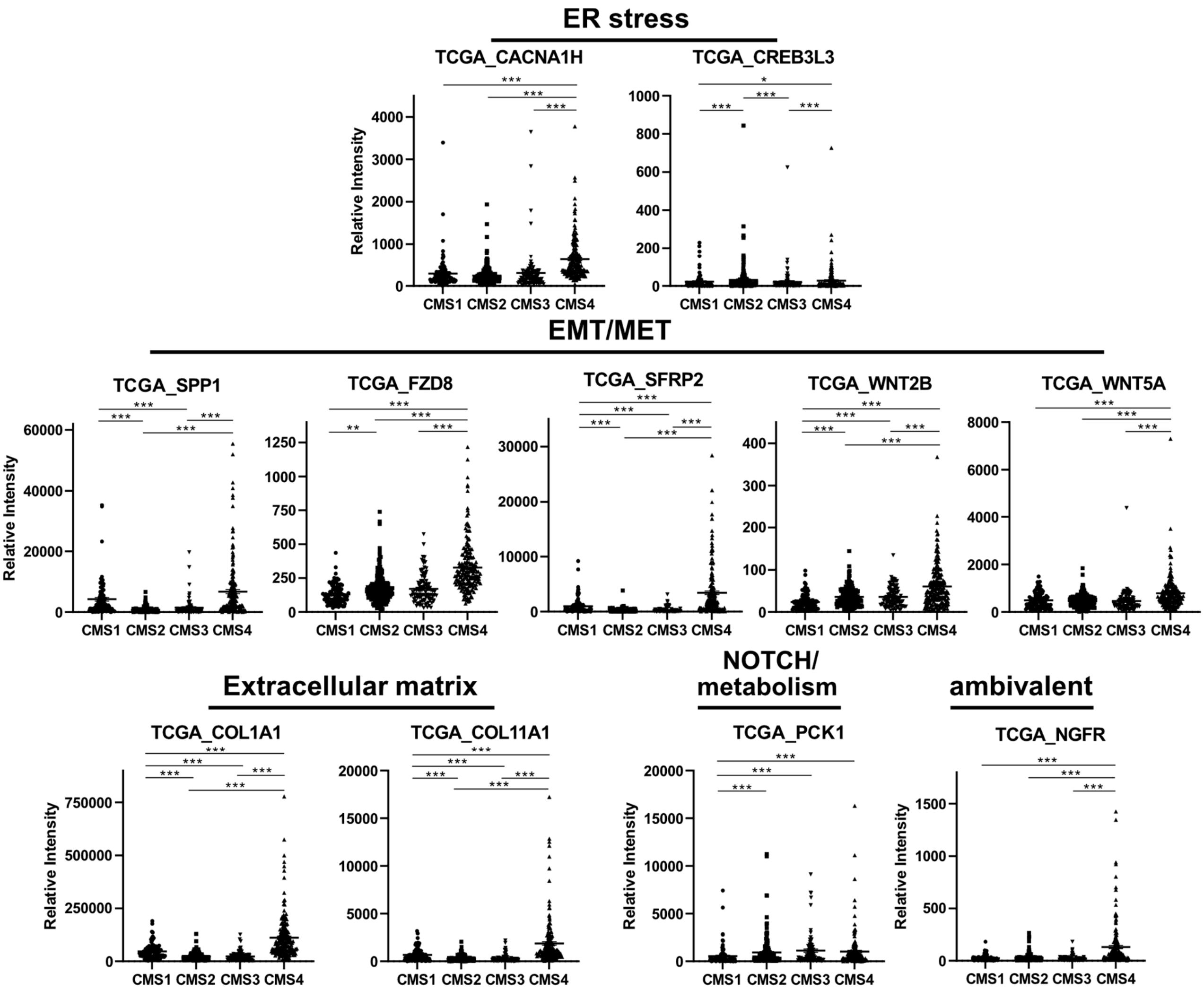

Figure S5. CMS group association of the confirmed signature genes in the TCGA (The Cancer Genome Atlas) Colorectal Adenocarcinoma data set. Associated cellular programs/pathways for each gene are indicated. Significance levels were calculated with Mann-Whitney test. *, $p<0.05$; **, $p<0.01$; ***, $p<0.001$; ns, not significant.

Table S1.

Recruitment centers
Charité Universitätsmedizin Berlin, Charitéplatz 1, 10117 Berlin
Onkologie Donauwörth (MVZ), Neudegger Alle, 86609 Donauwörth
Universitätsklinikum Essen, Hufelandstraße 55, 45147 Essen
Hämatoonkologische Tagesklinik (HOT) Landshut, Achdorfer Weg 5, 84036 Landshut
LMU Klinikum, University of Munich, Marchioninistraße 15, 81377 Munich
Klinikum rechts der Isar, Technical University Munich, Ismaninger Straße 22, 81675 Munich

Table S6. Signature genes in the REVEAL cohort with similar expression trend in two other CRC data sets. Avg expr, average expression (log_2); FC, fold change; P_{adj}, adjusted p value.

**GSE131418 (P (*n*=333) vs M (liver, *n*=137))**							
**gene**	**Log_2 FC**	**avg expr**	***p* value**	**P_{adj}**	**FC**	**%change**	**program/pathway/function**
COL11A1	-1.03	3.99	9.348×10^{-60}	3.552×10^{-59}	0.49	-51.03	ECM modulating/ related
COL1A1	-1.68	4.48	4.214×10^{-19}	1.001×10^{-18}	0.31	-68.79	ECM modulating/ related
FZD8	-0.75	6.91	5.625×10^{-36}	1.527×10^{-35}	0.59	-40.54	EMT/MET / WNT
SFRP2	-0.4	7.41	1.721×10^{-15}	3.27×10^{-15}	0.76	-24.21	EMT/MET / WNT
SPP1	0.14	8.45	0.00023	0.00026	1.10	10.19	EMT/MET / WNT
WNT2B	-1.63	8.12	3.251×10^{-84}	2.059×10^{-83}	0.32	-67.69	EMT/MET / WNT
WNT5A	-1.13	7.19	2.094×10^{-41}	6.63×10^{-41}	0.46	-54.31	EMT/MET / WNT
CACNA1H	-1.66	9.34	1.373×10^{-69}	6.521×10^{-69}	0.32	-68.36	ER stress, inhibitor of proliferation
CREB3L3	0.39	3.13	5.444×10^{-13}	7.956×10^{-13}	1.31	31.04	ER stress, transcription factor
NGFR	1.8	2.84	6.287×10^{-112}	1.194×10^{-110}	3.48	248.22	conflicting; tumor suppressor CRC
PCK1	0.71	3.35	3.697×10^{-9}	4.683×10^{-9}	1.64	63.58	NOTCH, metabolism

**GSE81582 (P (*n*=23) vs M (*n*=19))**							
**gene**	**Log_2 FC**	**avg expr**	***p* value**	**P_{adj}**	**FC**	**%change**	**program/pathway/function**
COL11A1	-1.76	7.61	0.0056	0.01071	0.30	-70.48	ECM modulating/ related
COL1A1	-0.36	10.34	0.34	0.36	0.78	-22.08	ECM modulating/ related
FZD8	-0.51	7.53	0.0003	0.0012	0.70	-29.78	EMT/MET / WNT
SFRP2	-2.44	6.43	0.0003	0.0012	0.18	-81.57	EMT/MET / WNT
SPP1	1.6	10.38	0.0026	0.006195	3.03	203.14	EMT/MET / WNT
WNT2B	-0.27	5.00	0.0087	0.014	0.83	-17.07	EMT/MET / WNT
WNT5A	-0.24	4.83	0.00072	0.0023	0.85	-15.33	EMT/MET / WNT
CACNA1H	-0.33	6.08	0.025	0.037	0.80	-20.45	ER stress, inhibitor of proliferation
CREB3L3	0.81	5.98	0.00014	0.00086	1.75	75.32	ER stress, transcription factor
NGFR	0.16	5.91	0.178	0.23	1.12	11.73	conflicting; tumor suppressor CRC
PCK1	1.26	8.47	0.069	0.093	2.39	139.50	NOTCH, metabolism
